# Supplementary material for: A fault-intrusion-tolerant system and deadline-aware algorithm for scheduling scientific workflow in the cloud
Source: PeerJ Comput Sci. 2021 Nov 2;7:e747. doi: 10.7717/peerj-cs.747 (PMC8576554; doi:10.7717/peerj-cs.747)
Supplement: Supplemental Information 6 [file peerj-cs-07-747-s006.zip › Myproject1.1/docs/org/cloudbus/cloudsim/network/TopologicalLink.html]

TopologicalLink


JavaScript is disabled on your browser.


- Overview
- Package
- Class
- Use
- Tree
- Deprecated
- Index
- Help

- Prev Class
- Next Class

- Frames
- No Frames

- All Classes

- Summary:
- Nested |
- Field |
- Constr |
- Method

- Detail:
- Field |
- Constr |
- Method


org.cloudbus.cloudsim.network

## Class TopologicalLink

- java.lang.Object
- - org.cloudbus.cloudsim.network.TopologicalLink

- ---

    

  ```
  public class TopologicalLink
  extends java.lang.Object
  ```

  This class represents an link (edge) from an graph

  Since:
  :   CloudSim Toolkit 1.0

- - ### Constructor Summary

    Constructors

    Constructor and Description

    `TopologicalLink(int srcNode,
    int destNode,
    float delay,
    float bw)`

    creates an new link-object
  - ### Method Summary

    Methods

    | Modifier and Type | Method and Description |
    | --- | --- |
    | `int` | `getDestNodeID()` return the node-ID from the DestNode |
    | `float` | `getLinkBw()` return the link-bw of the defined linke |
    | `float` | `getLinkDelay()` return the link-delay of the defined linke |
    | `int` | `getSrcNodeID()` returns the node-ID from the SrcNode |

    - ### Methods inherited from class java.lang.Object

      `clone, equals, finalize, getClass, hashCode, notify, notifyAll, toString, wait, wait, wait`

- - ### Constructor Detail


    - #### TopologicalLink

      ```
      public TopologicalLink(int srcNode,
                     int destNode,
                     float delay,
                     float bw)
      ```

      creates an new link-object
  - ### Method Detail


    - #### getSrcNodeID

      ```
      public int getSrcNodeID()
      ```

      returns the node-ID from the SrcNode

      Returns:
      :   nodeID


    - #### getDestNodeID

      ```
      public int getDestNodeID()
      ```

      return the node-ID from the DestNode

      Returns:
      :   nodeID


    - #### getLinkDelay

      ```
      public float getLinkDelay()
      ```

      return the link-delay of the defined linke

      Returns:
      :   the delay-amount


    - #### getLinkBw

      ```
      public float getLinkBw()
      ```

      return the link-bw of the defined linke

      Returns:
      :   the bw


- Overview
- Package
- Class
- Use
- Tree
- Deprecated
- Index
- Help

- Prev Class
- Next Class

- Frames
- No Frames

- All Classes

- Summary:
- Nested |
- Field |
- Constr |
- Method

- Detail:
- Field |
- Constr |
- Method
